# Should Society Allow Research Ethics Boards to Be Run As For-Profit Enterprises?

**DOI:** 10.1371/journal.pmed.0030309

**Published:** 2006-07-25

**Authors:** Ezekiel J Emanuel, Trudo Lemmens, Carl Elliot

## Abstract

Background to the debate: An important mechanism for protecting human research participants is the prior approval of a clinical study by a research ethics board, known in the United States as an institutional review board (IRB). Traditionally, IRBs have been run by volunteer committees of scientists and clinicians working in the academic medical centers where the studies they review are being carried out. However, for-profit organizations are increasingly being hired to conduct ethics reviews. Proponents of for-profit IRBs argue that these IRBs are just as capable as academic IRBs at providing high-quality ethics reviews. Critics argue that for-profit IRBs have a conflict of interest because they generate their income from clients who have a direct financial interest in obtaining approval.

## Abstract 2

Emanuel argues that for-profit ethics review boards can provide high quality reviews, but Lemmens and Elliot believe that such boards have a conflict of interest.

## Ezekiel J. Emanuel's Viewpoint: Let's Dump the Outdated Ideology of “For-Profit Bad, Not-for-Profit Good”

It is commonly thought that for-profit companies are necessarily worse than not-for-profit organizations doing similar activities. According to this thinking, for-profit companies are heartless, place profits over people, quality, or integrity, and are inherently exploitative. For-profit prisons must be worse than state-run prisons; for-profit health plans are worse than not-for-profit health plans; and surely for-profit auto manufacturers are worse than not-for-profit car makers.

This is a quaint notion. In prisons, we care about security and the respectful treatment and rehabilitation of the prisoners; in health plans we care about the quality of health care delivered to patients and the overall health of the people. Profit status is a tax designation, not a quality indicator. At best, profit status is a crude approximation—a proxy measure—for what we really care about: security and rehabilitation, or quality health care. What we should really focus on then is not the ideology of profit status but these substantive outcome data.

The same is true for IRBs. What we should focus on is not their tax status but the data regarding their quality. Are the IRBs evaluating protocols according to the ethical standards for clinical research? Are they ensuring that researchers are using reliable and valid scientific methods and selecting research participants fairly? Are the risks and benefits of the research reasonable? Does the informed consent document lucidly inform the patient without voluminous, superfluous, or distracting details? In addition, we want the IRB to carefully monitor the implementation of the protocol, especially monitoring for adverse events.

Why might we think that for-profit IRBs do these functions poorly? Perhaps being for-profit, they need to woo business, and so they are less independent of their clients, less inclined to be critical, and more inclined to overlook ethical problems.

But these potential difficulties are not unique to for-profit IRBs. Researchers who sit on not-for-profit, academic IRBs are evaluating their colleagues' research protocols, so these IRB members also have ties that may compromise their independence and critical evaluations. Furthermore, many academics tend to view IRB service as an uncompensated burden, which is not conducive to careful review work. Academic medical centers and their researchers also have their own financial interests in getting research protocols passed. They get money—as well as access to new drugs and prestige—for conducting the research. Many not-for-profit IRBs are also charging drug and device companies for review of their research protocols, and their rates are comparable to the rates charged by for-profit IRBs.

The crucial question is whether an IRB, regardless of its tax status, is performing at a high level of quality. Unfortunately, there are no validated quality indicators for IRB function, and no head-to-head comparison has been made of the performance of for-profit and not-for-profit IRBs. However, to evaluate the quality of IRB function we have some approximate indicators such as regulatory compliance, accreditation, knowledge sharing, and internal quality assurance practices.

In recent years the US's Office for Human Research Protections (OHRP) has suspended, at least temporarily, the research being conducted at a number of major medical centers—including Johns Hopkins, Duke University, Rush University, University of Colorado, and University of Rochester. Furthermore, unexpected deaths in research participants that have captured the public eye have occurred at some of these same institutions—Johns Hopkins and University of Rochester—as well as at the University of Pennsylvania and Case Western Reserve University. The suspension of research by OHRP and the unexpected death of relatively healthy research participants are not perfect indicators of poor studies or poor IRB performance, but neither can they be dismissed as irrelevant. All of these prominent cases have occurred at not-for-profit academic institutions.

The first IRBs to receive “full accreditation”—the highest level of accreditation awarded by the Association for the Accreditation of Human Research Protection Programs—were one for-profit IRB, Western Institutional Review Board (WIRB), and one not-for-profit IRB, University of Iowa. And so it continued: of the first eight organizations to receive full accreditation, four were for-profit IRBs.

At least 26 academic institutions outsource all or part of their protocol review to for-profit IRBs. And in recent years, the OHRP has called upon WIRB to re-review protocols and revamp the IRB processes and procedures at not-for-profit academic institutions where the OHRP had temporarily suspended research. Calling upon WIRB constitutes a vote of confidence by federal regulators that at least this one for-profit entity provides high-quality IRB review, and has something to teach the not-for-profit academic centers about institutionalizing quality IRB review.

Furthermore, OHRP has never suspended a for-profit IRB. And, the US Food and Drug Administration (FDA) has issued only one warning letter to a for-profit IRB compared to hundreds to IRBs at not-for-profit institutions.

For-profit IRBs have made contributions to the common good. For example, WIRB has established a program allowing IRB administrators from developing countries in Latin America, Southeast Asia, South Asia, and other places to visit their location for six months of training in running an IRB. So far, 24 fellows have been trained. How many not-for-profit IRBs have established programs to improve protocol review either in the US or other countries using their own money—not grants from the National Institutes of Health?

Making a distinction about the quality and merits of IRBs based on tax status is an antiquated piece of ideology reminiscent of Orwell's *Animal Farm*—“for-profit bad, not-for-profit good.” The distinction obscures what we should care about and directly measure—the quality of IRB review of protocols and the monitoring of the safety of research participants. There are absolutely no data showing that either independence of reviewers or quality outcomes are correlated with an IRB's profit status, and there is some real evidence in the regulatory actions and accreditation that the quality of for-profits is as good as or better than many of their not-for-profit academic counterparts. Since we are supposed to be researchers and driven by data not emotion, let's give up the crude ideology and stick to the data.

## Trudo Lemmens and Carl Elliot's Viewpoint: Commercial IRBs Have a Fundamental Conflict of Interest

Like it or not, research on humans has become a commercial enterprise. Most clinical trials have moved from academic settings to specialized contract research organizations (CROs), which contract with the pharmaceutical and biotechnology industries. Whereas 63% of clinical trials were still being undertaken in academic settings in 1994, a mere 26% of such trials remained there in 2004 [ 1].

It is widely recognized that the commercialization of medical research creates serious conflicts of interest [ 2–5]. What is often overlooked is that IRB review, which is often expected to provide protection against such conflicts, has also become commercialized. Some industry-funded trials are reviewed by in-house IRBs set up and funded by pharmaceutical companies or CROs themselves; others are reviewed by commercial, for-profit IRBs [ 6]. Of course, commercial IRBs are not an entirely new phenomenon. The oldest and most successful IRB, WIRB, has been in business since 1968. What is new about commercial IRBs is their extraordinary reach. According to its founder, WIRB is now responsible for the review of more than half of all new drug submissions to the FDA [ 7]. Several universities and hospitals also outsource ethics review to commercial IRBs.

Yet commercial IRBs have a fundamental conflict of interest [5,8–11]. They are in a client–provider business relationship with the commercial entities whose studies they review. Because commercial IRBs generate their income from clients with a direct financial interest in obtaining approval, they are affected by the very problem they are expected to curtail. The financial interests involved are huge. Pharmaceutical companies pay CROs for their speed and efficiency. Clinical trials are a crucial step in the drug development process. Any delay in approval by an IRB affects the sponsor's profit margins.

The countries that have allowed these private IRBs to flourish have also failed to regulate them carefully. Neither the US nor Canada has placed any serious restrictions on the establishment of new IRBs. Although an IRB registry has recently been set up for federally funded research in the US, and although the FDA and Health Canada sporadically inspect IRBs involved in the review of clinical trials, they do not have formal registration and approval processes for IRBs. Anyone who can bring together five people, including a community representative, a physician, a lawyer and an “ethicist,” can set up shop and start competing for business.

Moreover, regulations in those countries fail to prevent CROs from selecting the IRB least likely to reject the trial or delay approval by imposing too many restrictions. If one IRB is too stringent, they can simply go to the one next door.

Free market advocates will argue that the research industry has an interest in promoting quality IRB review. In theory, sound ethical review could prevent lawsuits, and commercial IRBs could use ethical review as a marketing tool. In fact, however, commercial IRBs market their speed and efficiency, and lawsuits are still relatively rare. Most are settled out of court, with the records sealed from public view. Moreover, the costs of these lawsuits pale in comparison with the profit gained by bringing a new drug faster to market. For multinational pharmaceutical companies, litigation is a manageable cost of doing business.

Commercial IRBs fill a regulatory vacuum in countries that lack a governmentally organized system of ethics review. Yet, as *Bloomberg Markets* reported, several commercial IRBs have approved and been involved in overseeing controversial research practices. SFBC International, the largest CRO in the United States, has recruited undocumented immigrants to a converted motel in Miami and paid them to enroll in trials overseen by an unlicensed medical director [ 7]. Some of SFBC International's clinical trials were approved by a now dismantled commercial IRB owned by the wife of an SFBC International vice president [ 7]. To oversee the research conducted at the Fabre Research Clinic, the clinic's owner founded a private IRB that had his business partner and lawyer as members. The clinic was eventually shut down by the FDA after an investigation into the death of a research participant and more than ten years after the agency first flagged several serious research irregularities [ 12]. In another article, *Bloomberg Markets* reported that a Canadian affiliate of SFBC International, Montreal's Anapharm, is currently being investigated by the Canadian drug regulatory authorities after several human research participants were infected with tuberculosis. Research participants had been submitted to basic medical screening, according to the report, but not to any specific tests for tuberculosis, even though the trial involved an immunosuppressant drug [ 13].

In 1999, Swiss authorities were alerted that a CRO in the Canton Basel was recruiting individuals from Eastern Europe and asylum seekers as research participants for Swiss clinical trials. An investigation conducted by a special working group for the drug regulatory agency revealed troubling consent procedures, such as consent forms being in languages the trial participants did not understand. The principal investigator for the clinical trials, who combined the position of CRO director with that of local director of the commercial IRB that approved the research, was not licensed to practice medicine [ 14].

At a time when commercial interests threaten the safety of research participants and the integrity of medical research, it is remarkable that North American regulatory agencies have not seen any problem with entrusting the rights and well-being of human research participants to a lightly regulated commercial enterprise. In the wake of the Swiss scandal, the authorities in Basel introduced new regulations requiring registration and regulatory approval of IRBs. IRBs also received exclusive jurisdiction, making it impossible to shop for the most lenient IRB. These new regulations, under which no commercial IRBs were approved, were upheld by the Swiss Supreme Court. The court argued that research ethics committees fulfill a public function with a direct mandate from the state [ 15, 16]. It is time for regulatory authorities in Canada and the US to follow suit. The protection of research participants is a critically important public mandate, and it merits a truly independent regulatory structure.

## Emmanuel's Response to Lemmens and Elliot's Viewpoint

I agree with Lemmens and Elliot that “the protection of research subjects is a critically important public mandate, and it merits a truly independent regulatory structure.” That is where the agreement ends.

Lemmens and Elliot claim that “commercial IRBs have a fundamental conflict of interest.” But IRBs at academic medical centers have even greater conflicts of interest [ 17]. Both commercial and academic IRBs have a financial conflict of interest, especially since increasingly academic IRBs are charging competitive prices for their services, making them indistinguishable from commercial IRBs. Academic IRBs have the additional conflict that the researchers being reviewed are colleagues of the IRB members. And they have yet a further conflict since the institution wants and needs the commercial research in order to gain access to new drugs and devices to enhance its reputation as innovative.

Lemmens and Elliot conflate CROs and commercial IRBs, trying to tar the reputation of commercial IRBs with unsavory practices of CROs. Furthermore, when all the hyperbole is ignored, these authors base all their objections on just three anecdotes, rather than on rigorous scientific data. That the owners/operators of SFCB, Fabre Research Clinic, and a research center in Switzerland have been accused of unscrupulous practices hardly constitutes an indictment of all commercial IRBs. Using similar logic one might indict all not-for-profit IRBs because of the documented poor performance of academic IRBs that had to be suspended at Johns Hopkins, Duke University, University of Colorado, University of Rochester, etc.

Lemmens and Elliot marshal absolutely no scientific data to show that any of the major commercial IRBs in the US—including WIRB, Schulman, Chesapeake, Essex, and Copernicus—are performing poorly. The authors cite no studies, for example, to show that the quality of the reviews by these IRBs is poor or that these IRBs have approved unethical studies. I do not know whether these IRBs are all good, but if Lemmens and Elliot's broad indictment leveled against all commercial IRBs is valid, they surely should provide data about the poor quality of the major commercial IRBs, since these account for the majority of reviews done in the commercial sector. Otherwise, the charges appear to be good headlines, but are baseless.

Finally, despite their rhetoric, Lemmens and Elliot do not actually call for the prohibition of commercial IRBs. They want more regulatory oversight. Yet, I am unaware of their having delineated a comprehensive proposal describing such an oversight system. With Wood and Grady, I have proposed a system of regional ethics organizations that would review and monitor research protocols, educate researchers on the ethics of human research, and develop policies in controversial areas, such as paying participants [ 18]. Such organizations would completely eliminate conflicts of interest, financial and otherwise, between the reviewing organizations and researchers. Maybe this is something Lemmens and Elliot can agree with? If not, let's hear their positive proposal for a “truly independent regulatory structure.”

## Lemmens and Elliott's Response to Emanuel's Viewpoint

Not too long ago, an article on the protection of sick and vulnerable research participants that compared the funding of IRBs to the funding of for-profit jails would have been read as satire. In North America today, however, where medical research happily converges with consumer capitalism, even bioethicists believe that the market ultimately works for justice. Do we need to point out that the problem with the consumerist model for protecting research participants is not, as Emanuel suggests, its “tax status”? The problem is that commercial IRBs are paid in full by the very companies conducting the research. What is more, those companies are free to shop around for any IRB whose reviews they find congenial. Research participants who are worried that they may face death or injury in a study sponsored by a pharmaceutical company are unlikely to feel more secure knowing that their safety has been entrusted to a panel of paid experts whose financial livelihood depends on a company paycheck.

Acknowledging that no good data exist to compare commercial and university IRBs, Emanuel nevertheless exhorts us to “stick to the data” anyway, recommending the example of WIRB, a commercial IRB that earned $20 million in 2004, and whose president was Emanuel's research collaborator (as he acknowledges in his competing interests statement). Yet part of the data he omits from his enthusiastic recommendation comes from *Bloomberg Markets*, which reported in December that WIRB “oversaw tests in California and Georgia in the 1990s for which doctors were criminally charged and jailed for lying to the FDA and endangering the lives of trial participants. No action was taken against WIRB” (p. 37 of [ 7]). The same report revealed that WIRB had settled a lawsuit after it approved a placebo-controlled trial for a Genentech psoriasis drug in which a patient was severely injured, that it had drawn criticism from the FDA in inspection visits, and that on one occasion, when reviewing protocols for Johns Hopkins University, it reversed a previous decision under pressure from a clinical sponsor, using a panel dominated by alternate members.

It is rarely, if ever, possible to know whether financial incentives have improperly influenced a member of an IRB. This uncertainty is the reason for rules about conflicts of interest—to prevent people from being placed in situations where they are likely to be improperly influenced. We agree with Emanuel that academic IRBs are marred by many of the same problems facing for-profit IRBs, including conflicts of interest [ 6, 19]. But the problems of academic IRBs do not make those of for-profit IRBs disappear. The proper solution is to clean up the conflicts of interest, not to institute a replacement in which such conflicts are built right into the system.

## Abbreviations

CRO: contract research organizations
FDA: US Food and Drug Administration
IRB: institutional review board
OHRP: Office for Human Research Protections
WIRB: Western Institutional Review Board

## References

[pmed-0030309-b1] Steinbrook M (2005). Gag clauses in clinical-trial agreements. N Engl J Med.

[pmed-0030309-b2] Krimsky S (2003). Science in the private interest: Has the lure of profits corrupted biomedical research?.

[pmed-0030309-b3] Bekelman J, Li Y, Gross CP (2003). Scope and impact of financial conflicts of interest in biomedical research: A systematic review. JAMA.

[pmed-0030309-b4] Bodenheimer T (2000). Uneasy alliance—Clinical investigators and the pharmaceutical industry. N Engl J Med.

[pmed-0030309-b5] Lemmens T (2004). Leopards in the temple: Restoring scientific integrity to the commercialized research scene. J Law Med Ethics.

[pmed-0030309-b6] Lemmens T, Freedman B (2000). Ethics review for sale? Conflict of interest and commercial research review boards. Milbank Q.

[pmed-0030309-b7] Evans D, Smith M, Willen L (2005). Big pharma's shameful secret. Bloomberg Mark.

[pmed-0030309-b8] Francis L, Spece RG, Shimm DS, Buchanan AE (1996). IRBs and conflicts of interest.

[pmed-0030309-b9] Federman DD, Hanna KE, Lyman Rodriguez L (2003). Responsible research: A systems approach to protecting research participants.

[pmed-0030309-b10] Department of Health and Human Services Office of Inspector General (1998). Institutional review boards: The emergence of independent boards.

[pmed-0030309-b11] Elliott C, Lemmens T (2005). Ethics for sale: For-profit ethical review coming to a clinical trial near you. Slate. http://www.slate.com/id/2132187.

[pmed-0030309-b12] Evans D (2005). Garry Polsgrove's last battle. Bloomberg Markets.

[pmed-0030309-b13] Evans D (2005). SFBC drug testers have tuberculosis after exposure at centre. Bloomberg News.

[pmed-0030309-b14] Groupe de Travail “Réglementation des Essais Cliniques” (2000). Rapport final à l'intention de l'Office Intercantonal de Contrôle des Médicaments.

[pmed-0030309-b15] Swiss Bundesgerichts Second Public Law Division (2003).

[pmed-0030309-b16] Jost A (2003). Arrêt du 4 juillet de la IIème cour de droit public du Tribunal Fédéral, Freiburger Ethik-Kommission International c. Bâle-Campagne. Rev Suisse Droit Santé.

[pmed-0030309-b17] Emanuel EJ, Wood A, Fleischman A, Bowen A, Getz KA (2004). Oversight of human participants research: Identifying problems to evaluate reform proposals. Ann Intern Med.

[pmed-0030309-b18] Wood A, Grady C, Emanuel EJ (2004). Regional ethics organizations for protection of human research participants. Nat Med.

[pmed-0030309-b19] Boyd EA, Cho MK, Bero LA (2003). Financial conflict-of-interest policies in clinical research: Issues for clinical investigators. Acad Med.

