# Correction: Should Society Allow Research Ethics Boards to Be Run As For-Profit Enterprises?

**DOI:** 10.1371/journal.pmed.0030391

**Published:** 2006-09-12

**Authors:** Ezekiel J Emanuel, Trudo Lemmens, Carl Elliott

In *PLoS Medicine*, volume 3, issue 7: DOI: 10.1371/journal.pmed.0030309

The name Carl Elliot was misspelled; the correct spelling is Carl Elliott.

